# Idiopathic pleuroparenchymal fibroelastosis: consideration of a clinicopathological entity in a series of Japanese patients

**DOI:** 10.1186/1471-2466-12-72

**Published:** 2012-12-05

**Authors:** Hideki Kusagaya, Yutaro Nakamura, Masato Kono, Yusuke Kaida, Shigeki Kuroishi, Noriyuki Enomoto, Tomoyuki Fujisawa, Naoki Koshimizu, Koshi Yokomura, Naoki Inui, Takafumi Suda, Thomas V Colby, Kingo Chida

**Affiliations:** 1 Second Division, Department of Internal Medicine, Hamamatsu University School of Medicine, Hamamatsu, Japan; 2 Department of Laboratory Medicine, Hamamatsu University School of Medicine, Hamamatsu, Japan; 3 Department of Clinical Pharmacology and Therapeutics, Hamamatsu University School of Medicine, Hamamatsu, Japan; 4 Department of Laboratory Medicine/Pathology, Mayo Clinic Arizona, Scottsdale, Arizona, USA

**Keywords:** Idiopathic interstitial lung disease, Pleural fibrosis, Fibroelastosis, Pleuroparenchymal fibroelastosis

## Abstract

**Background:**

Idiopathic pleuroparenchymal fibroelastosis (IPPFE) is a recently reported group of disorders characterized by fibrotic thickening of the pleural and subpleural parenchyma predominantly in the upper lobes. We report five Japanese cases fulfilling the criteria of IPPFE and address whether it should be considered a separate clinicopathologic entity. And this study was an attempt to identify features in common between IPPFE and previously described idiopathic upper lobe fibrosis (IPUF), allowing IPPFE to be considered as a distinct entity in our Japanese series.

**Methods:**

Five consecutive cases of idiopathic interstitial lung disease confirmed as IPPFE by surgical lung biopsy were studied.

**Results:**

There were four males and one female, aged 70±2.76 yr. No associated disorder or presumed cause was found in any case. Lung function tests found a restrictive ventilatory defect (4/5) and/or impairment of DLco (4/5). Chest X-ray showed marked apical pleural thickening in all cases. Computed tomography of the chest in all cases mainly showed intense pleural thickening and volume loss associated with evidence of fibrosis, predominantly in the upper lobes. In all cases in this study, markedly thickened visceral pleura and prominent subpleural fibrosis characterized by both elastic tissue and dense collagen were clearly shown. All cases were alive at the last follow-up, 17.6±13.59 months after diagnosis; however, all had deteriorated both clinically and radiologically.

**Conclusions:**

IPPFE deserves to be defined as a separate, original clinicopathologic entity owing to its uniformity and IPPFE has some features in common with previously described idiopathic upper lobe fibrosis (IPUF). Our limited experience with a cohort of 5 subjects suggests that IPPFE can be rapidly progressive.

## Background

Idiopathic pleuroparenchymal fibroelastosis (IPPFE) is a recently reported rare disorder characterized by fibrotic thickening of the pleural and subpleural parenchyma predominantly in the upper lobes [1]; as such IPPFE is distinct from other types of idiopathic interstitial pneumonia [2]. Since 1992 several reports of upper lobe-predominant pulmonary fibrosis (Idiopathic upper lobe fibrosis/IPUF) have been reported in the Japanese literature [3-9] and some of these cases share features with IPPFE. Herein we report five Japanese cases of IPPFE, and describe the clinical, radiologic and evolutionary profile of IPPFE in a retrospective series in order to determine if IPPFE represents a separate clinicopathologic entity and to clarify its relationship with IPUF.

## Methods

The subjects included 5 patients fulfilling criteria IPPFE diagnosed by surgical lung biopsy [1] (SLB) from May 2009 to Sep 2011 at Hamamatsu University Hospital and affiliated hospitals in Japan. Lung biopsies were performed by video-assisted thoracoscopy, and specimens were obtained from 2 or more lobes in every case. We used the pathological criteria for the diagnosis of IPPFE as follows: (1) intense fibrosis of the visceral pleura; (2) prominent, homogenous, subpleural fibroelastosis; (3) sparing of the parenchyma distant from the pleura; (4) mild, patchy lymphoplasmocytic infiltrates; and (5) small numbers of fibroblastic foci present. All of our reported cases met all of the criteria. This criteria is based on the paper by Frankel et al. [1]. Surgical lung specimens were also reviewed by an expert pulmonary pathologist (T.V.C.). This study was approved by the Institutional Review Board of Hamamatsu University School of Medicine. Clinical data were obtained from case medical records. Laboratory findings and pulmonary function test results at the time of SLB were also recorded.

## Results

### Clinical features and biology

Table 1 shows the clinical characteristics and laboratory data. All patients were more than 60 years of age. There were no current smokers, and only one patient was an ex-smoker. Four out of five were classified as underweight by body mass index (BMI). The main presenting symptom was dyspnea. There was no significant family or occupational history. Case 1 had a history of gingival cancer, and had chemotherapy. Mild lymphocytosis was found on BAL in one case. Examination and cultures of lung tissue for bacteria, mycobacteria, fungi, parasites, and viruses were negative in all cases. KL-6 was elevated in two cases and normal in three. Interestingly, the level of SP-D was higher than the normal range in all the cases.

**Table 1:** Clinical characteristics and laboratory data

	**Case.1**	**Case.2**	**Case.3**	**Case.4**	**Case.5**
Age (yr)/sex	70, male	74, female	67, male	67, male	72, male
Smoking status	Ex-smoker	Never-smoker	Never-smoker	Never-smoker	Never-smoker
BMI	18.7	17.5	15.9	17.9	17.0
Symptom	Dyspnea	Dyspnea	Cough	Dyspnea	Dyspnea
Crackles	+	-	-	-	+
BAL lym (%)	8.6	0	16	1	ND
neu (%)	0.6	0	1.0	8	ND
eos (%)	0	1	0	3	ND
KL-6 (U/ml)	410	604	437	1000	469
SP-D (ng/ml)	204	131	129	437	133
RF (U/ml)	54	-	4	1	16
ANA	-	×40	×40	-	-
PaO_2_ (Torr)	82	94.4	95.8	83.0	82.0
Prognosis	Survive 12 months	Survive 6 months	Survive 44 months	Survive 10 months	Survive 16 months

Abbreviations: **BMI**, body mass index, **BAL**, broncho-alveolar lavage, **KL-6**, Sialylated carbohydrate antigen KL-6, **Sp-D**, Surfactant Protein D, **RF***,* rheumatoid factor, **ANA**, antinuclear antibody.

### Pulmonary function test

All cases underwent a spirometry test and measurement of total lung capacity (TLC). A restrictive ventilatory defect defined by %FVC < 80% was found in 4 cases. In addition, four cases had impairment of DLco (Table 2).

**Table 2:** Pulmonary function test data

	**Case.1**	**Case.2**	**Case.3**	**Case.4**	**Case.5**
TLC (L), %predicted (%)	4.09 (79.4)	2.90 (79.0)	5.97 (107.4)	3.69 (72.1)	2.9 (56.9)
FVC (L), %predicted (%)	2.50 (74.1)	1.41 (74.1)	3.24 (93.6)	2.14 (63.7)	2.12 (64.0)
FEV1 (L), %predicted (%)	2.06 (77.5)	1.33 (87.5)	3.20 (101.6)	2.11 (79.0)	1.67 (64.3)
FEV1.0/FVC	90.4	95	96.7	96.3	96.5
FRC (L), %predicted (%)	3.0 (75.2)	2.1 (101)	3.87 (85.6)	2.61 (67.3)	2.14 (51.8)
RV (L), %predicted (%)	1.97 (121.6)	1.42 (87.1)	2.82 (154.1)	1.55 (100)	1.38 (84.1)
RV/TLC (%), %predicted (%)	48.17 (120.2)	48.97 (145.1)	47.2 (120.7)	42.0 (107.2)	47.59 (111.2)
DLco (mL/min/mmHg), %predicted (%)	9.41 (74.2)	9.94 (76.8)	10.55 (71.3)	12.51 (96.1)	6.12 (55.1)
DLco/VA (mL/min/mmHg), %predicted (%)	3.04 (69.1)	4.42 (103.3)	2.10 (47.1)	4.42 (98.4)	2.78 (64.5)

Abbreviations: **TLC**, Total lung capacity, **FVC**, Forced vital capacity, **FEV**, Forced expiratory volume, **FRC**, Functional residual capacity, **RV** Residual volume, **DLco**, Diffusing capacity for carbon monoxide, **DLco/VA**, Diffusing capacity divided by the alveolar volume.

### Radiological features

**Figure 1:**
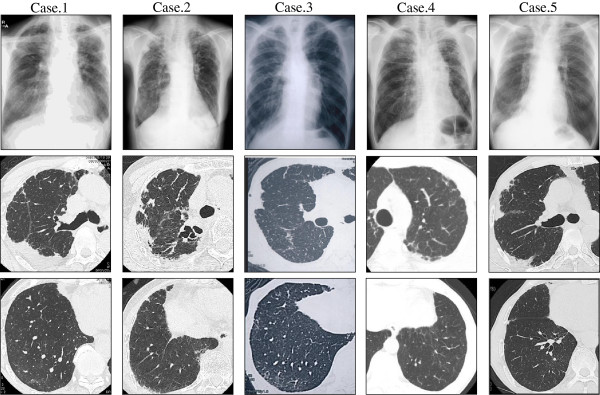
**Chest X-ray and Chest CT.** Chest radiographs in all cases showed marked apical pleural thickening. High-resolution CT (HRCT) showed upper lobe volume loss, architectural distortion, traction bronchiectasis, and reticular abnormalities. There was no honeycombing.

Chest radiographs in all cases showed marked apical pleural thickening and superior hilar retraction (Figure 1). Right lung is predominantly affected in case 2, 3, and 5, left lung in case 4. High-resolution CT (HRCT) showed intense pleural thickening associated with evidence of fibrosis. In Case 1 and 2, upper lobe volume loss, architectural distortion and traction bronchiectasis were also prominent.

### Pathological features

**Figure 2:**
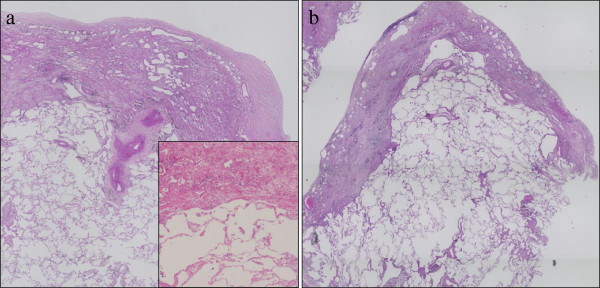
**Histopathological Findings (hematoxylin-eosin).** Surgical lung biopsy specimen at low power (**a**:case 1, **b**:case 5) and high power (a, inset) showed markedly thickened visceral pleura and prominent subpleural fibrosis characterized by abnormal increase of elastic tissue and dense collagen. Abrupt transition to normal parenchyma was also seen. Parenchyma distant from the pleura was spared.

**Figure 3:**
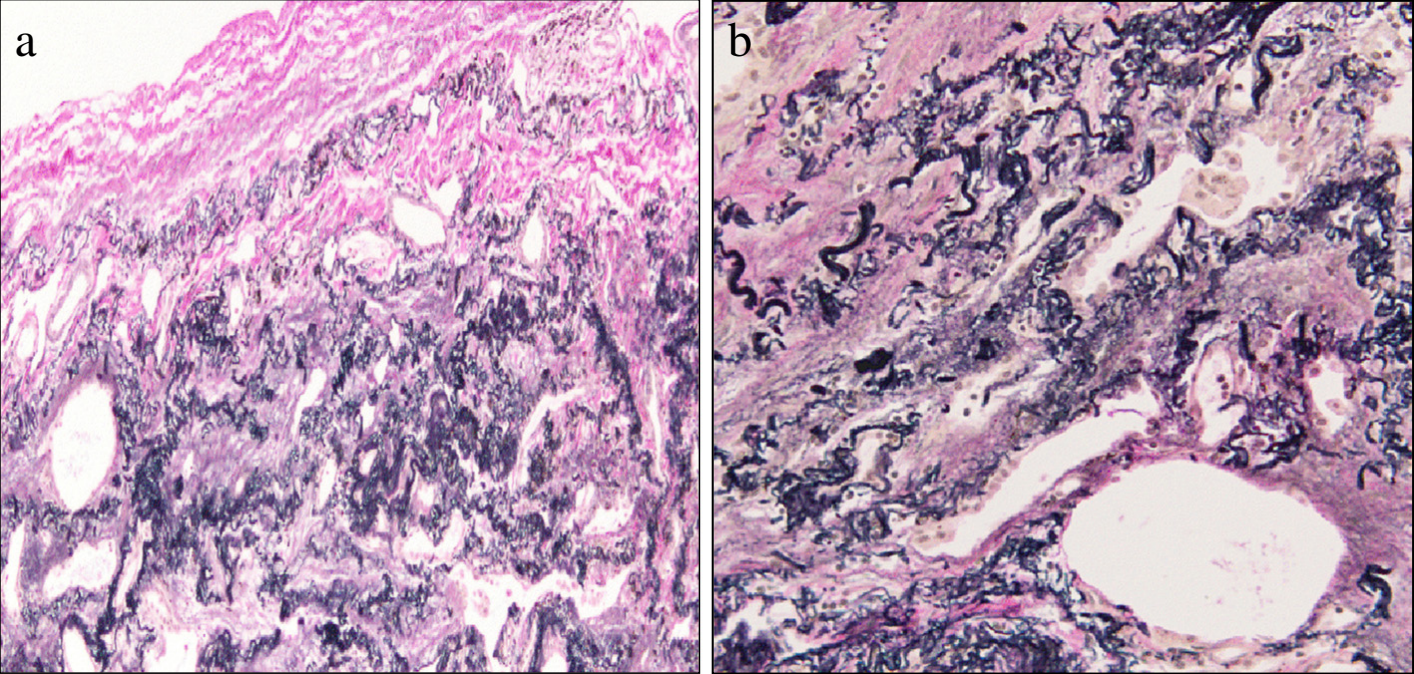
**Histopathological Findings (Elastic Van Gieson).** Surgical lung biopsy specimen at low power (**a**) and high power (**b**) demonstrated an abundance of short, amorphous elastic fibers (Case 1).

In all cases in this study, the histopathological findings fitted those previously described for IPPFE. Markedly thickened visceral pleura and prominent subpleural fibrosis characterized by both elastic tissue and dense collagen were clearly shown (Figures 2a, b and 3a, b). The border between the fibroelastosis and the underlying normal lung parenchyma was abrupt, and the parenchyma distant from the pleura was spared (Figure 2a). Fibroblastic foci were rarely noted at the leading edge of the fibrosis. Inflammation was variable and primarily consisted of small aggregates of lymphocytes. Asbestos bodies were absent.

### Treatment and clinical outcome

**Figure 4:**
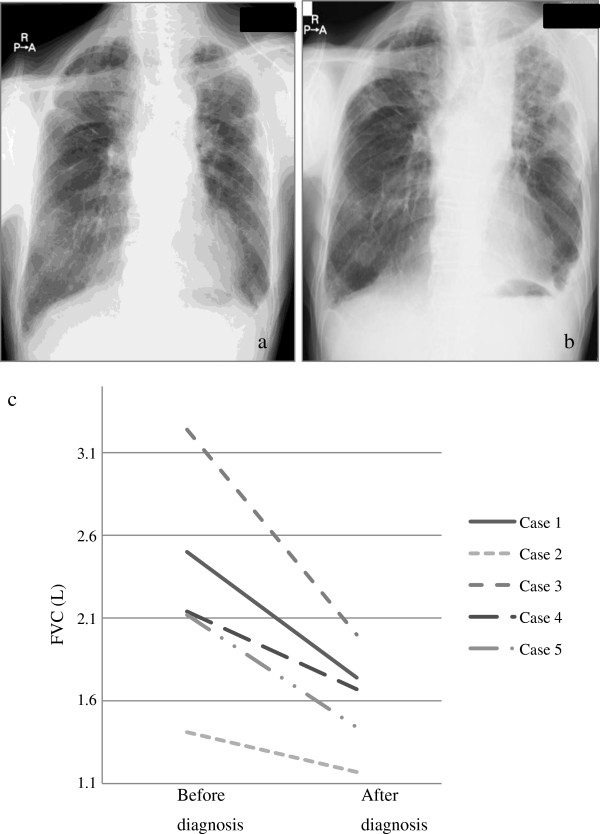
**Clinical Course of the cases.** Case 1 radiologically deteriorated a year after diagnosis (**a**, **b**). Other cases present similar behavior. (**c**) Decrease in FVC was confirmed in all cases during the follow up. The median follow up was 12.1 (range 4.37-22.2) months.

All cases initially had no treatment. The mean duration of follow-up after the pathologic diagnosis was 12.1 months (range 4.4 - 22). The total duration of follow-up from the onset of the symptoms to the last follow-up was 45.2 months (range 7–83). All patients were alive at the last follow-up (Table 2), but all had disease progression according to clinical and lung function tests, especially in terms of %FVC (Figure 4).

## Discussion

It is clear that IPPFE is not a new pulmonary disease. As Reddy et al [10] reported, it has previously received other names, such as idiopathic upper lobe fibrosis (IPUF), and has more frequently been described in the Japanese literature [3-5,9] and may be more common in the Japanese. IPPFE has been defined as a distinct entity in the English literature by Frankel et al.; a unique clinical presentation and precise characteristic pathologic criteria are described [1]. Our study was an attempt to identify features in common between these two entities, allowing IPPFE to be considered distinct as it applies to our Japanese series.

The clinical presentation and the findings of the lung function tests were characteristic of restrictive interstitial lung disease. Radiological features were also characteristic, including intense pleural thickening associated with evidence of fibrosis, most striking pattern in the upper lobes with volume loss and architectural distortion. The upper lobes were always more severely involved, with involvement of the lower lobes being absent or less marked. Piciucchi and colleagues reported that one case had honeycombing on HRCT findings in their cases [11]. In our series, no patients had honeycombing. In all the reports describing PPFE, eight out of total 25 cases (32%) had a history of malignancy. Among those cases, four cases had treatment with BMT [12] and those cases presented with histological evidence of obliterative bronchiolitis, which was not a feature in those with idiopathic presentation of PPFE, suggesting that bone marrow transplantation is a potential etiology at least in a subgroup of cases with pleuroparenchymal fibroelastosis. In our series, Case 1 had a history of gingival cancer treated with surgery followed by adjuvant chemotherapy. However, no cases had BMT, and other cases never had malignancy.

In terms of therapy and prognosis, previous cases of IPPFE showed a 40% mortality rate. In addition, many cases gradually deteriorated. In our series, consistent with previous reports, although no death occurred, all the cases clinically and functionally deteriorated even in a relatively short follow-up period. There are no therapeutic options available for IPPFE except for supportive care and ultimately lung transplantation. Regarding the mechanism of elastosis, it has not been determined whether fibroelastosis occurred by overproduction of elastin or impairment of degradation. Pirfenidone is an antifibrotic drug, currently used only for idiopathic pulmonary fibrosis (IPF) [13-15], which inhibits fibrotic factors, most notably TGFβ. As a consequence, downstream synthesis of extracellular matrix proteins, such as fibronectin, elastin and collagen, is reduced [16,17]. Hence, it may potentially be beneficial for cases of IPPFE.

There are several case reports of idiopathic, upper lobe-predominant fibrosis in the Japanese language literature [3-6,9]. Among those reports, it has been described that the thinness of the body was a characteristic of the disease. Consistent with these reports, our cases showed low BMI. However, there are no data on BMI or body weight of patients with IPPFE in reports from outside of Japan. Radiologically upper lobe predominance is associated with both IPUF and IPPFE. As both IPUF and IPPFE are relatively rare and only recently described, there are no guidelines or criteria as to how extensive the opacities in the middle and lower lobes can be. In terms of other aspects of the disease, such as possible occurrence of pneumothorax, infection and slow progression of the disease, IPPFE and IPUF seem to be within the same spectrum of disease. Although IPUF patients with surgical lung biopsy or autopsy were reported in some Japanese literature, there are no pathological criterias as IPUF. But in all those cases, intraalveolar fibrosis and subpleural elastosis were noted. Those findings are same to the pathological findings with IPPFE. Interestingly, the specimens from some patients shows UIP like pattern in the lower lobe. Amitani et al. [9] strictly defined the lesion is in the upper lobe, but the report from Shiota et al. [6] include the patients with UIP like lesion. Reddy [10] also mentioned patients with PPFE had the fibrosis in the lower lobe. Further studies are needed what is the possible lesion in mid-lower lobe. Finally, pulmonary surfactant protein D (SP-D) was elevated in all cases in our series. It is expressed in alveolar type II and bronchiolar epithelial cells and is secreted into alveoli and conducting airways [18,19]. Our data suggest that those epithelial cells may play a role in the fibrotic process of the diseases.

There are some limitations to the present study. First, there were selection and recall biases because this was a retrospective study. Since the current authors’ institutions are regional referral centers for diffuse lung disease, the proportion and severity of the disease reported here may differ substantially from those found in the community. Second, the sample size was too small to determine the precise prevalence and clinical characteristics of IPPFE. Prospective studies incorporating larger series are necessary to verify these results.

## Conclusions

This study shows that the clinical, radiological and pathological presentation of IPPFE is remarkably uniform in cases with apparently idiopathic lung disease. We thus consider that idiopathic PPFE deserves to be defined as a distinct clinicopathological syndrome largely overlapping with previously described IPUF. It should be distinguished from other types of interstitial lung disease, especially because it is highly progressive.

### Consent

Written informed consent was obtained from the patient for publication of this case report and accompanying images. A copy of the written consent is available for review by the Editor-in-Chief of this journal.

## Abbreviations

BMI: body mass index; BAL: broncho-alveolar lavage; KL-6: sialylated carbohydrate antigen KL-6; Sp-D: *surfactant protein D*; RF: rheumatoid factor; ANA: antinuclear antibody; TLC: total lung capacity; FVC: forced vital capacity; FEV: forced expiratory volume; FRC: functional residual capacity; RV: residual volume; DLco: diffusing capacity for carbon monoxide; DLco/VA: diffusing capacity divided by the alveolar volume.

## Competing interests

All authors declare that we have no competing interests.

## Authors’ contributions

HK: Conception and design, Data collection, Data analysis and interpretation, Manuscript writing. YN: Conception and design, Data analysis and interpretation, Manuscript writing, Final approve of manuscript. MK: Data collection. YK: Data collection. TF: Data collection. NE: Data collection. HS: Data collection. NK: Data collection. KY: Data collection. TS: Data analysis. T.V.C: Conception and design, Data analysis and interpretation, Manuscript writing. KC: Conception and design, Administrative support, Data analysis and interpretation. All authors read and approved the final manuscript.

## Pre-publication history

The pre-publication history for this paper can be accessed here:

http://www.biomedcentral.com/1471-2466/12/72/prepub
